# Education, early screening and treatment of STIs could reduce infertility among women in Kenya

**Published:** 2017-06

**Authors:** SM Musundi

**Affiliations:** Institute of Women, Gender and Development Studies, Egerton University, Njoro, Kenya

**Keywords:** education, female infertility, government, involuntary childlessness, Kenya, prevalence, STIs

## Abstract

In Kenya, sexually transmitted infections (STIs) such as Chlamydia trachomatis, Neisseria gonorrhoea, HIV, herpes simplex virus type 2 (HSV-2), syphilis and trichomoniasis tend to be prevalent, especially in women. Further, the research shows that women who test positive for STIs (other than HIV), have little knowledge of these infections. Of particular concern, is that there has been little attention on the part of government to educate the general public about STIs, yet these diseases can have devastating consequences on women’s and men’s health. In women, STIs can produce sequelae such as tubal infertility. To help reduce female factor infertility, the Kenya government should conduct a nationwide campaign to educate the public about the importance of screening and treatment of STIs.

## Introduction

According to the World Health Organization (WHO, 2012), low-income countries have the greatest burden of sexually transmitted infections (STIs). In 2008, there were an estimated 92.6 million new cases of four curable STIs (gonorrhoea, chlamydia, syphilis and trichomoniasis) among adults aged 15–49 years in sub-Saharan Africa (WHO, 2012). It is also estimated that in 2012, this region had the highest prevalence (32%) of herpes simplex virus type 2 (HSV-2), a sexually transmitted viral infection that is incurable (Looker et al., 2015). These data, along with country-specific studies across the region, show that women are those most affected by STIs (Weiss et al., 2001; Ombelet et al., 2008a; Morhason-Bello, 2014; Looker et al., 2015; Osei, 2016).

Extant research also indicates that if left untreated, STIs can have devastating health consequences in both women and men (WHO, 2012). In women, STIs can lead to sequelae such as tubal factor infertility (Ombelet et al., 2008; Dhont et al., 2011; WHO, 2012; Apari et al., 2014). WHO (2012) notes that, “in sub-Saharan Africa, untreated genital infection may be the cause of up to 85% of infertility among women seeking infertility care”. STI prevalence is often compounded by the asymptomatic nature of these diseases, which contributes to delayed diagnosis and intervention (Ombelet et al., 2008).

In Kenya, as in other regions of the world, STIs tend to be prevalent, especially in women (Weiss et al., 2001; Kohli et al., 2013; Masese et al., 2013; Vandenhoudt et al., 2013; Musyoki et al., 2015; Maina et al., 2016; Okango et al., 2016; Masha et al., 2017). According to the Kenya AIDS Indicator Survey (KAIS 2007) - which documents the national prevalence and incidence of HIV and STIs in Kenya - an estimated 35% of study participants aged 15-64 years had genital herpes (HSV-2). Of this population, 42% were women and 26% men (Mugo et al., 2011). In the KAIS 2012 report, it is estimated that 0.9% of persons aged 15-64 years old reported having a STI in the 12 months prior to the study. Compared to men, women reported more cases of symptoms that are often associated with these infections. For example, 6.2% of women reported having had an abnormal vaginal discharge compared to 1.5% of men who reported a penile discharge. Further, 3.8% of women stated that they had had genital ulcers compared with 1.6% of men who reported having had this condition (NASCOP, 2014).

While the KAIS 2012 report shows that Kenya has a low STI prevalence overall, these data are based entirely on self-report and may be subject to recall bias. Furthermore, given that STIs tend to be asymptomatic, and that women can at times be unable to distinguish between ‘normal’ and ‘abnormal’ vaginal discharge for example, it is likely that respondents could have unintentionally over- or underreported these infections. Small cross-sectional studies that have examined special categories of women (e.g. pregnant women, women who engage in transactional sex and HIV-positive women) as well as women in the general population in Kenya, suggest that there are disparities in the STI disease burden within these groups and that prevalence is highest in high-risk groups.

In a cross-sectional study involving 202 women attending antenatal clinic at the Kilifi County Hospital in the coastal town of Kilifi in Kenya, Masha et al. (2017) found that one in five women had a curable STI. These infections included Chlamydia trachomatis (14.9%), gonorrhoea (1.0%), Tricho- monas vaginalis (7.4%) bacterial vaginosis (19.3%) and genital ulcers (2/5%). In another cross-sectional study of women attending a family planning clinic at the Kenyatta National Hospital in Nairobi, Kenya, Maina et al. (2016), found that 13 per cent of female respondents tested positive for Chlamydia trachomatis, with the highest prevalence being in women aged 25-29 and lowest in those aged 45-49 years. Of those who tested positive, 64 per cent were asymptomatic. Interestingly, most of the participants who had C. trachomatis were married and had only one sexual partner in the 12 months prior to the study. In a third study involving women attending antenatal care at two hospital sites in Nairobi, Kohli et al. (2013) found prevalence of genital chlamydia to be at 6 per cent. As with the Maina et al. study, the majority of patients who tested positive for chlamydia in the Kohli et al. study, were also asymptomatic and most had little or no knowledge of this infection.

Contrary to this research, some studies show that research participants with prior STI/HIV education demonstrate an awareness of these infections (Luchters et al., 2008). For example, in a cross- sectional study involving 506 female sex workers (FSW) in Mombasa, Kenya, Luchters et al. (2008) found that 145 (28.7%) FSW who attended STI/HIV peer-education sessions had higher levels of knowledge of these infections (e.g. symptoms and prevention methods) and reported more consistent condom use with clients than their counterparts who did not attend these sessions. Despite having knowledge of STIs/HIV however, these FSW still had high rates of HIV (29.6%) and other STIs particularly bacterial vaginosis (44%), candida infection (23%), Trichomonas vaginalis (14%) and genital ulcers (13%) although these rates were lower than in FSW with no STI/HIV training. One of the factors that could have contributed to the sustained prevalence of STI/HIV among the trained cohort was that they had inconsistent condom use with their spouses or boyfriends.

Similar high rates of STI prevalence have been found in other studies involving women who are characterized as ‘high-risk’(Fonck et al., 2001; Chohan et al., 2009; Masese et al., 2013; Vandenhoudt et al., 2013; Jespers et al., 2014; Musyoki et al., 2015; Winston et al., 2015). For example, Winston et al.’s cross-sectional study (2015) of 200 youth living on the streets in Eldoret, Kenya, shows that female respondents had a high rate of infections including herpes simplex virus type 2 (35%), C. trachomatis (16%), gonorrhoea (15%), HIV (15%), trichomoniasis (15%) and syphilis (6%). According to the researchers, transactional sex, drug and alcohol use, having multiple sexual partners and a prior STI increased female respondents’ propensity of acquiring a sexually transmitted infection (Winston et al., 2015).

In another cross-sectional study consisting of 481 female sex workers (FSW) in Kisumu, Kenya, Vandenhoudt and colleagues (2013) found that the majority of participants tested positive for HIV (56.5%) and other STIs including HSV-2 (83.8%), active syphilis (3.4%), gonorrhoea (5.9%), C. trachomatis (3.4%), genital ulcers (5.6%), Trichomonas vaginalis (13.6%), and bacterial vaginosis (27%). Participants’ HIV status was associated with a number of factors including: STI status in the past one year, marital status, ethnicity, number of years doing sex work, money received for last sex act and STI treatment in the past year. The high rate of HSV-2 among HIV-infected FSW in Kisumu is consistent with prior studies that have shown a significant association between HSV-2 and HIV infection and transmission (Reynolds et al., 2003; Brown et al., 2007; Okango et al., 2015; Daniels et al., 2016).

Despite the overwhelming research evidence suggesting that women and girls in Kenya are disproportionately affected by STIs apart from HIV, and that they have little knowledge about these diseases, there appears to be little effort from the government to sensitize the public about these infections and the importance of early screening and treatment for STIs. Indeed, one rarely comes across messages in the media from Ministry of Health that address this issue. There has also been no public campaign linking STIs to reproductive issues like infertility.

In fact, infertility in and of itself, has received little attention as a sexual and reproductive health concern (Ndegwa, 2016; Gerrits et al., 2017) despite the fact that Kenya is estimated to have an overall sub-fertility rate of 26.1 per cent with 50 per cent being attributed to tubal factor infertility and 15 per cent to male factors (Murage et al., 2011). It is surprising that much of the public education around STIs has focused on HIV/AIDS prevention and care while overlooking other STIs, yet, research evidence in Kenya and elsewhere in sub-Saharan Africa shows a strong association between other STIs and HIV acquisition and transmission (Dhont et al., 2011; Mugo et al., 2011; Barnabas and Celum, 2012; Kakaire et al., 2015; Looker et al., 2015; Musyoki et al., 2015; Okango et al., 2015; Daniels et al., 2016; Masha et al., 2017). Further, some studies suggest that HSV-2 in particular, can accelerate HIV disease progression in persons who are HIV-infected (Lingappa et al., 2001; Roxby et al., 2011). Given the health implications that STIs pose on people’s lives, one wonders why these infections receive so little attention in Kenya. With limited dissemination of information surrounding STIs other than HIV, how is the general public supposed to know about these infections?

It is very likely that a higher number of women than what has been previously documented in various studies in Kenya are living with curable STIs unknowingly, since these diseases tend to be asymptomatic, resulting in individuals’ failure to seek treatment (Daniels et al., 2016; Maina et al., 2016). Women who do not visit their obstetrician/gynaecologist or family planning clinic routinely, are less likely to be screened and /or treated for STIs and may be unaware of the potential impact of these infections on their health outcome, particularly fertility. Indeed, recent studies on infertility conducted elsewhere on the African sub-continent have found that respondents have little knowledge of the link between STIs and infertility (Dhont et al., 2011; Lamaran et al., 2016).

Arguably, the Kenyan public has a right to be informed about STIs other than HIV, and it is the responsibility of the government to ensure that people have access to this information. It would be unfair for couples and/or individuals to miss the opportunity to have children simply because they were un- informed about the implication of STIs on fertility, and consequently, failed to seek treatment for these infections. In African culture, children are highly valued and couples who are unable to reproduce face a lot of stigma from the wider society (Dyer et al., 2005; Dyer, 2007; Fledderjohann, 2012; Tabong and Adongo, 2013a, 2013b). Therefore, to spare many couples the agony of becoming involuntarily childless and having to deal with social stigma and other problems emanating from their childlessness (Van Balen and Bos, 2009; Dhont et al., 2011; Fledderjohann, 2012; Lamaran et al., 2016; Osei, 2016;), the government should encourage youth and adults who are sexually active to undergo routine screening for STIs other than HIV. Furthermore, the government should subsidize the cost of screening and treatment of STIs (just as it has done with HIV), since these costs may hinder the unemployed or poor from accessing services (Masese et al., 2013; Maina et al., 2016). Such an intervention may go a long way in helping to reduce the burden of STIs in the general population, and in turn, lessen the prevalence of female infertility in Kenya.
